# Leucocyte nadir as a marker for chemotherapy efficacy in node-positive breast cancer treated with adjuvant CMF

**DOI:** 10.1038/sj.bjc.6690594

**Published:** 1999-08

**Authors:** P Poikonen, T Saarto, J Lundin, H Joensuu, C Blomqvist

**Affiliations:** Department of Oncology, Helsinki University Central Hospital, Helsinki, FIN-00029 HYKS, Finland; HUCH Clinical Research Institute, Helsinki, Finland

**Keywords:** adjuvant chemotherapy, breast neoplasms, CMF, leucopenia

## Abstract

The purpose of this study was to examine the association between the leucocyte nadir and prognosis in breast cancer patients receiving adjuvant chemotherapy consisting of cyclophosphamide, methotrexate and fluorouracil (CMF). Three hundred and sixty-eight patients with node-positive breast cancer without distant metastases were treated with six cycles of adjuvant CMF. Some patients (*n* = 60) also received tamoxifen. All patients underwent surgery and received radiotherapy to the axillary and supraclavicular lymph nodes and the chest wall. The effect of leucopenia caused by CMF on distant disease-free survival (DDFS) and overall survival (OS) was assessed. A low leucocyte nadir during the chemotherapy was associated with a long DDFS in univariate analysis when tested as a continuous variable (the relative risk (RR) 1.3, 95% confidence interval (CI) 1.04–1.06, *P* = 0.02). Similarly, when the leucocyte nadir count was divided into tertiles, the patients who had the highest nadir values during the six-cycle treatment had worst outcome (RR 1.6, 95% CI 1.07–2.5, *P* = 0.02). However, in a multivariate analysis only the number of affected lymph nodes, tumour size, progesterone receptor status, surgical procedure, age and adjuvant tamoxifen therapy retained prognostic significance, whereas the leucocyte nadir count did not. A low leucocyte nadir during the adjuvant CMF chemotherapy is associated with favourable DDFS and it may be a useful biological marker for chemotherapy efficacy. © 1999 Cancer Research Campaign


*British Journal of Cancer* (1999) **80**(11), 1763–1766
© 1999 Cancer Research Campaign
Article no. bjoc.1998.0594

### Leucocyte nadir as a marker for chemotherapy efficacy in node-positive breast cancer treated with adjuvant CMF

**P Poikonen[1], T Saarto[1], J Lundin[2], H Joensuu[1] and C Blomqvist[1]**

[1]Department of Oncology, Helsinki University Central Hospital, PO Box 180, FIN-00029 HYKS, Helsinki, Finland; [2]HUCH Clinical Research Institute, Helsinki, Finland

**Summary** The purpose of this study was to examine the association between the leucocyte nadir and prognosis in breast cancer patients receiving adjuvant chemotherapy consisting of cyclophosphamide, methotrexate and fluorouracil (CMF). Three hundred and sixty-eight patients with node-positive breast cancer without distant metastases were treated with six cycles of adjuvant CMF. Some patients ($n$ = 60) also received tamoxifen. All patients underwent surgery and received radiotherapy to the axillary and supraclavicular lymph nodes and the chest wall. The effect of leucopenia caused by CMF on distant disease-free survival (DDFS) and overall survival (OS) was assessed. A low leucocyte nadir during the chemotherapy was associated with a long DDFS in univariate analysis when tested as a continuous variable (the relative risk (RR) 1.3, 95% confidence interval (CI) 1.04–1.06, $P$ = 0.02). Similarly, when the leucocyte nadir count was divided into tertiles, the patients who had the highest nadir values during the six-cycle treatment had worst outcome (RR 1.6, 95% CI 1.07–2.5, $P$ = 0.02). However, in a multivariate analysis only the number of affected lymph nodes, tumour size, progesterone receptor status, surgical procedure, age and adjuvant tamoxifen therapy retained prognostic significance, whereas the leucocyte nadir count did not. A low leucocyte nadir during the adjuvant CMF chemotherapy is associated with favourable DDFS and it may be a useful biological marker for chemotherapy efficacy.

**Keywords:** adjuvant chemotherapy; breast neoplasms; CMF; leucopenia

Dose intensity in chemotherapy is defined as the amount of drug delivered per unit time. Bonadonna and Valgussa found in 1981 that disease-free survival was shorter in node-positive breast cancer patients who received a reduced dose of adjuvant chemotherapy consisting of cyclophosphamide, methotrexate and fluorouracil (CMF) than in those who received full or nearly full dose (Bonadonna and Valagussa, 1981) Similar findings have been published in other retrospective studies in early and advanced breast cancer (Rodriguez et al, 1981; Tormey et al, 1983; Howell et al, 1984; Senn et al, 1984; Ang et al, 1989; Pronzato et al, 1989) while in some studies no significant correlation between the given dose and survival benefit was found (Ahmann et al, 1982; Glucksberg et al, 1982; Redmond et al, 1983; Mouridsen et al, 1984; Velez-Garcia et al, 1987). These studies have been criticized due to the bias inherent in this kind of retrospective analysis (Redmond et al, 1983). Patients with a larger tumour burden may tolerate chemotherapy poorly due to the presence of occult bone marrow metastases and may receive a lower total dose. This may create a false association between a low dose intensity and poor outcome.

Only a few controlled prospective studies of the dose–response relationship in breast cancer have been published, and the results have not been consistent. Only one study has shown evidence of benefit in the adjuvant setting for conventional doses as compared to doses lower than conventional (Wood et al, 1994). Two controlled studies have failed to demonstrate any dose–response relationship (Fumoleau et al, 1993; Fisher et al, 1997). Only one of these studies, studying escalated versus conventional doses (Fisher et al, 1997) has, however, been sufficiently large to detect the expected difference of a few per cents between the treatment groups.

Haematological toxicity is the most important dose-limiting toxicity and often the reason for a dose reduction. By studying the association between experienced toxicity and outcome, the bias associated with the retrospective studies can be circumvented. We found in a previous study that a low leucocyte nadir during adjuvant chemotherapy comprising cyclophosphamide, doxorubicin and oral fthorafur (CAFt) in stage II and III breast cancer patients associated with a long distant disease-free (DDFS) and overall survival (OS) (Saarto et al, 1997). Similar findings were also reported in a study by Ahman et al (1982).

CMF is perhaps the most widely used chemotherapy combination in adjuvant treatment of breast cancer. We, therefore, wanted to study whether high haematological toxicity was associated with a better outcome also with the CMF regimen.

### MATERIALS AND METHODS

### Patients

The study population comprised of 368 consecutive pre- and perimenopausal women with primary T1–4 histologically proven node-positive breast cancer, without distant metastases. All patients were treated with post-operative adjuvant CMF between

*Received 11 March 1998*
*Revised 3 October 1998*
*Accepted 20 November 1998*

*Correspondence:* to C Blomqvist

1987 and 1993 in Helsinki University Hospital, Department of Oncology .

All patients underwent surgery with axillary clearance and total mastectomy or breast-conserving resection. Patients were treated with adjuvant chemotherapy , which consisted of six cycles of cyclophosphamide (600 mg m$^{-2}$), methotrexate (40 mg m$^{-2}$) and fluorouracil (600 mg m$^{-2}$) administered intravenously on day 1 at 3 -week intervals. A total of 363 patients underwent post-operative irradiation, and 63 received adjuvant tamoxifen. The majority of patients who received radiotherapy had irradiation to the regional lymph nodes and operative scar or the remaining breast with megavoltage irradiation (45–50 Gy in 18–25 fractions) after the second or the third cycle of chemotherapy , or after six cycles of chemotherapy .

Staging investigations included clinical investigation, liver enzymes, chest X-ray , liver scintigraphy or ultrasound and bone scintigraphy . The white blood cell counts, haemoglobin and thrombocytes were investigated during chemotherapy , 1 day before treatment and approximately on day 16 of the cycle (s.d. 4.7). After treatment the patients were followed up at 3- to 4-month intervals for the first 2 years and thereafter at 6-month intervals for at least 5 years. After the fifth year of follow-up the control visits were performed once a year . The control examinations included clinical investigation, liver enzymes and serum creatinine at 3- to 6-month intervals. A bone scan, liver ultrasound and chest X-ray were performed only upon suspicion of recurrence. The median follow-up time was 68 months (range 5–126).

Data for 19 patients were excluded from the analyses because nine patients had disease progression during chemotherapy patients received fewer than four chemotherapy cycles, and one patient had missing data on leucocyte nadir values. Therefore, 349 patients were eligible for analysis. Six patients had missing data on the relative dose intensity . Ten patients with ot histology than ductal or lobular cancer were excluded from the prognostic analysis of the histological type.

### Calculation of haematological toxicity and leucocyte nadir

For analyses of the effect of haematological toxicity on DDFS an OS we used the minimum leucocyte count, which was the lowest leucocyte count measured during the entire course of chemotherapy .

### Definitions and calculation of doase intensity

The doses of each chemotherapy drug, the duration of treatment and the body surface area were extracted from the patient records. The absolute dose intensity was defined as the amount of drug administered per unit body surface area (mg m$^{-2}$) delivered per unit time (mg m$^{-2}$ week$^{-1}$). The relative dose intensity was calculated as the delivered dose intensity divided by the projected dose intensity , according to Longo et al (1991). The projected dose intensity is the total amount of drugs scheduled in the protocol, divided by the projected time schedule of the entire treatment. The delivered dose intensity represents the total amount of drug actually received, divided by the time taken for the therapy intensities for each drug were calculated for the total number of cycles (DI). Duration of treatment was defined as the interval (in weeks) between day 1 of the first cycle of chemotherapy and day 21 of the last given cycle.

For example, for a patient who received 450 mg m$^{-2}$ cyclophosphamide and 35 mg m$^{-2}$ methotrexate (five cycles during 22 weeks), the dose intensity of cyclophosphamide is ((450 mg m$^{-2}$ × 5) / 22 weeks) / 150 mg m$^{-2}$ / week = 0.68 = 68% and dose intensity of methotrexate is ((35 mg m$^{-2}$ × 5) / 22 weeks)/ 11.7 mg m$^{-2}$/week = 0.68 = 68%.

### Statistical methods

Life tables were calculated according to Kaplan–Meier (Kaplan and Meier , 1958). The statistical significance of the difference between the survival curves was calculated using the log-rank test (Peto et al, 1977) and Cox regression analysis (Cox, 1972). Only deaths due to breast cancer were scored as events in the analysis of OS. In the univariate and multivariate survival analysis the covariates were entered according to the categories presented in Tables 1 and 2. Multivariate survival analyses were performed by entering the leucocyte nadir as a continuous and a categorical variable. Covariates were selected in a backward stepwise fashion, with the use of the maximum-likelihood ratio. A *P*-value of 0.05 was adopted as the limit for inclusion of a covariate.

**Table 1** Cox univariate distant disease-free survival analysis in patients with node-positive breast cancer

| | *n* | RR | CI 95% | *P*-value |
|---|---|---|---|---|
| Leucocyte nadir tertiles | | | | |
| Lowest | 115 | 1 | | |
| Middle | 122 | 1.29 | 0.84–1.98 | 0.25 |
| Highest | 112 | 1.64 | 1.08–2.48 | 0.02 |
| Leucocyte nadir continuous | 349 | 1.31 | 1.04–1.64 | 0.02 |
| Axillary lymph nodes | | | | |
| 1–3 | 232 | 1 | | |
| > 3 | 106 | 2.56 | 1.82–3.59 | < 0.0001 |
| Tumour size | | | | |
| T1 | 139 | 1 | | |
| T2 | 169 | 2.55 | 1.69–3.84 | < 0.0001 |
| T3 | 33 | 4.17 | 2.44–7.14 | < 0.0001 |
| Progesterone receptor | | | | |
| Positive | 186 | 1 | | |
| Negative | 114 | 2.28 | 1.60–3.25 | < 0.0001 |
| Oestrogen receptor | | | | |
| Positive | 186 | 1 | | |
| Negative | 121 | 2.04 | 1.44–2.88 | < 0.0001 |
| Surgical procedure | | | | |
| Resection | 75 | 1 | | |
| Mastectomy | 274 | 2.72 | 1.59–4.64 | 0.0003 |
| Tamoxifen | | | | |
| No | 287 | 1 | | |
| Yes | 61 | 1.80 | 1.11–2.93 | 0.02 |
| Grade | | | | |
| 1 | 18 | 1 | | |
| 2 | 102 | 3.03 | 0.94–9.77 | 0.06 |
| 3 | 78 | 3.96 | 1.22–12.86 | 0.02 |
| Histologic type | | | | |
| Ductal | 302 | 1 | | |
| Lobular | 37 | 1.98 | 1.01–3.90 | 0.05 |
| Age | | | | |
| 35–49 | 271 | 1 | | |
| ≥ 50 | 47 | 1.13 | 0.69–1.85 | 0.62 |
| < 35 | 31 | 2.12 | 1.30–3.46 | 0.003 |

*© 1999 Cancer Research Campaign*

**Table 2** Multivariate distant disease-free survival analysis in 283 patients with node-positive breast cancer. Recurrent breast cancer was detected in 115 (41%) of these patients at the end of follow-up

| | RR | CI 95% | *P*-value |
|---|---|---|---|
| Axillary lymph nodes | | | |
| 1–3 | 1.00 | | |
| > 3 | 2.56 | 1.76–3.74 | < 0.0001 |
| Tumour size | | | |
| T1 | 1.00 | | |
| T2 | 2.02 | 1.28–3.16 | 0.002 |
| T3 | 2.66 | 1.45–4.88 | 0.002 |
| Progesterone receptor | | | |
| Positive | 1.00 | | |
| Negative | 2.20 | 1.48–3.26 | < 0.0001 |
| Surgical procedure | | | |
| Resection | 1.00 | | |
| Mastectomy | 3.23 | 1.72–6.09 | 0.0003 |
| Age | | | |
| 35–49 | 1.00 | | |
| ≥ 50 | 0.82 | 0.46–1.47 | NS |
| < 35 | 2.38 | 1.38–4.07 | 0.002 |
| Tamoxifen | | | |
| Yes | 1.00 | | |
| No | 2.11 | 1.20–3.72 | 0.009 |
| Leucocyte nadir tertiles | | | NS |
| Oestrogen receptor | | | NS |
| Histologic type | | | NS |
| Grade | | | NS |

NS, not significant.

### RESULTS

The median number of chemotherapy cycles given was six. The mean total dose of cyclophosphamide was 90% (range 42–104%) of the planned total dose, the mean total dose of methotrexate was 94% (range 36–123%) and that of fluorouracil 90% (range 47–103%). The mean relative dose intensity of cyclophosphamide was 85% (range 39–113%), methotrexate 88% (range 37–119%) and fluorouracil 84% (range 20–116%). The main reason for dose reduction was leucopenia (*n* = 45, 13%). Other such reasons were skin erythema due to radiotherapy and elevated liver enzymes. The main reason for a low dose intensity was a break in chemotherapy caused by radiotherapy. The mean leucocyte nadir value was 2.3 (s.d. 0.7, range, 0.6–5.1). The nadir leucocyte tertiles were less than 2.0, 2.0–2.7 and greater than 2.7. The overall dose intensity was similar in all leucocyte tertile groups (mean: 88%, 87% and 87% for the lowest, middle and highest third respectively)

### Univariate analysis

In univariate analyses there was a significant association between a low leucocyte nadir and a long DDFS (*P* = 0.02 both when analysed by the log-rank test for a trend as tertiles and when tested as a continuous variable by Cox regression analysis) (Figure 1). Other factors significantly associated with a long DDFS in a univariate analysis were a low number of metastatic lymph nodes, a small tumour size, positive oestrogen and progesterone receptor status, breast-conserving surgical procedure, adjuvant tamoxifen therapy, low differentiation grade, ductal histological type, and age between 35 and 49 (Table 1). The relative dose intensity was not significantly associated with DDFS. Patients with a low leucocyte nadir tended to have a longer OS in a univariate analysis

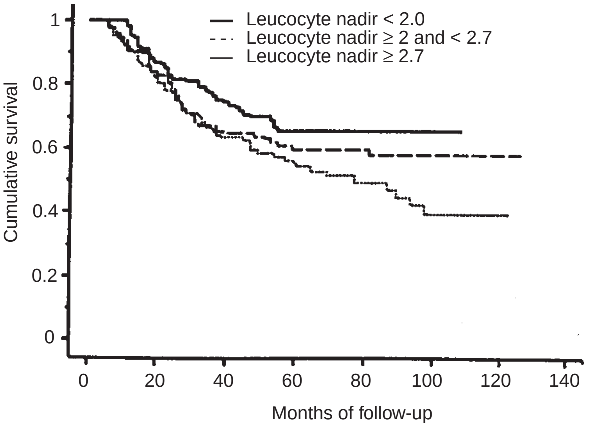

**Figure 1** Distant disease-free survival according to leucocyte tertiles in patients with node-positive breast cancer

(*P* = 0.06). When restricted to patients with a nadir value taken during days 9–14 (*n* = 99) the association between leucopenia and DDFS (risk ratio 1.56, *P* = 0.05) was stronger than in the entire series (risk ratio 1.31, *P* = 0.02)

### Multivariate analysis

All variables which were significantly associated to DDFS or OS in a univariate analysis were included in a multivariate analysis. The nodal status, tumour size, progesterone receptor status, the type of surgery (mastectomy or conservative resection), age and tamoxifen therapy were significantly associated with DDFS (Table 2), while the leucocyte nadir lost its significance. In analyses of OS the number of affected lymph nodes, tumour size, oestrogen and progesterone receptor status, the type of surgery and age were independent prognostic factors.

We also tested the correlation among different variables by $\chi^2$ method. The only variable which correlated with leucocyte nadir was age (*P* = 0.03). Patients aged 44 years or older had higher leucocyte nadir values than younger patients. Tumour size, nodal status, steroid receptor status, tamoxifen therapy, histological type, grade or surgical procedure had no correlation with leucocyte nadir.

### DISCUSSION

To our knowledge there are no published controlled trials testing the dose intensity of CMF in the adjuvant setting. In a recent study (Wood et al, 1994), however, low dose chemotherapy produced inferior results when compared to conventional dose cyclophosphamide, adriamycin and fluorouracil as adjuvant chemotherapy in node-positive breast cancer. On the other hand, in another adjuvant study with more than 2000 randomized patients even a fairly large increase in the dose intensity of cyclophosphamide resulted in only increased toxicity without any survival benefit (Fisher et al, 1997).

Several retrospective analyses of dose intensity of CMF in the adjuvant setting have been published, but these can be criticized for statistical bias. Patients with poor tolerance to chemotherapy, e.g. due to subclinical metastases and low performance status, may receive lower doses. This may create a false positive dose–response association. This is not likely the case with the

© 1999 Cancer Research Campaign present retrospective analysis of toxicity as a surrogate marker of dose effect, because patients who have leucopenia due to subclinical bone marrow metastases are expected to decrease the association between leucopenia and favourable DDFS.

Earlier we found an association between a low leucocyte nadir and a high overall survival rate among 211 breast cancer patients who were treated with doxorubicin-based adjuvant chemotherapy (CAFt) (Saarto et al, 1997). In the present study, a similar association was found for the CMF regimen, but the association was, however, relatively weak. There may be several explanations for this. The study population comprises patients who were treated with routine adjuvant CMF therapy, and the timing of leucocyte nadir tests varied. Actually, the association between DDFS and degree of leucopenia was stronger if the analyses were restricted to patients whose nadir values fell between days 9 and 14 of the cycle. Even if the association between the leucocyte nadir and outcome is due to individual differences in the treatment effect, the association is expected to be weak as compared to other prognostic variables. According to the results from the EBCTCG overview study the difference in 10-year recurrence-free survival was 25% between nodal negative and positive groups, while adjuvant CMF improved 10-year recurrence-free survival with only 8% (Anonymous, 1992).

The leucocyte nadir was not an independent prognostic factor in a multivariate survival analysis. We did not, however, find any significant correlation between the nadir and other prognostic variables. Most of the other known prognostic factors have a stronger effect on outcome than adjuvant chemotherapy. Thus, if the leucocyte nadir is used as a marker of chemotherapy effect, it is not expected to be as strong as other variables entered, and may, therefore, easily fall out of the final prognostic model.

In a recent study by Colleoni et al (1998), reductions larger than 35% in the dose administered, oral CMF adversely influenced outcome of breast cancer patients. The best outcome was in the intermediate dose group, which received 65–85% of the intended dose. Colleoni et al considered that it may be postulated that for adjuvant chemotherapy the clinically achieved effect, as evidenced by moderate toxicity, better correlates with survival than the total dose delivered.

The results of the present study suggest that leucopenia could be used as a biological indicator of an effective chemotherapy dose. Whether the outcome of those patients who do not experience leucopenia can be improved by dose-escalation can only be studied in a randomized trial.

### ACKNOWLEDGEMENTS

This investigation was supported by Amgen Oy.

### REFERENCES

Ahmann DL, O'Fallon JR, Scanlon PW, Payne WS, Bisel HF, Edmonson JH, Frytak S, Hahn RG, Ingle JN, Rubin J and Creagan ET (1982) A preliminary assessment of factors associated with recurrent disease in a surgical adjuvant clinical trial for patients with breast cancer with special emphasis on the aggressiveness of therapy. *Am J Clin Oncol* **5**: 371–381

Ang PT, Buzdar AU, Smith TL, Kau S and Hortobagyi GN (1989) Analysis of dose intensity in doxorubicin-containing adjuvant chemotherapy in stage II and III breast carcinoma. *J Clin Oncol* **7**: 1677–1684

Bonadonna G and Valagussa P (1981) Dose–response effect of adjuvant chemotherapy in breast cancer. *N Engl J Med* **304**: 10–15

Colleoni M, Price K, Castiglione-Gertch A, Goldhirch A and Coates A (1998) Dose–response effect of adjuvant cyclophosphamide, methotrexate, 5-fluorouracil (CMF) in node-positive breast cancer. *Eur J Cancer* **34**: 1693–1700

Cox D (1972) Regression models and life tables. *J R Statist Soc Ser* **B34**: 187–220

Early Breast Cancer Trialists' Collaborative Group (1992) Systemic treatment of early breast cancer by hormonal, cytotoxic, or immune therapy: 133 randomised trials involving 31 000 recurrences and 24 000 deaths among 75 000 women. *Lancet* **339**: 1–15

Fisher B, Anderson S, Wickerham DL, DeCillis A, Dimitrov N, Mamounas E, Wolmark N, Pugh R, Atkins JN, Meyers FJ, Abramson N, Wolter J, Bornstein RS, Levy L, Romond EH, Caggiano V, Grimaldi M, Jochimsen P and Deckers P (1997) Increased intensification and total dose of cyclophosphamide in a doxorubicin-cyclophosphamide regimen for the treatment of primary breast cancer: findings from National Surgical Adjuvant Breast and Bowel Project B-22. *J Clin Oncol* **15**: 1858–1869

Fumoleau P, Devaux Y, Vo VM, Kerbrat P, Fargeot P, Schraub S, Mihura J, Namer M and Mercier M (1993) Premenopausal patients with node-positive resectable breast cancer. Preliminary results of a randomised trial comparing 3 adjuvant regimens: FEC 50 × 6 cycles vs FEC 50 × 3 cycles vs FEC 75 × 3 cycles. The French Adjuvant Study Group. *Drugs* **2**: 38–45

Glucksberg H, Rivkin SE, Rasmussen S, Tranum B, Gad El, Mawla N, Costanzi J, Hoogstraten B, Athens J, Maloney T, McCracken J and Vaughn C (1982) Combination chemotherapy (CMFVP) versus L-phenylalanine mustard (L-PAM) for operable breast cancer with positive axillary nodes: a Southwest Oncology Group Study. *Cancer* **50**: 423–434

Howell A, Rubens R and Bush H (1984) A controlled trial of adjuvant chemotherapy with melphalan versus cyclophosphamide, methotrexate and fluorouracil for breast cancer. *Resent Results Cancer Res* **96**: 74–89

Kaplan E and Meier P (1958) Non-parametric estimation from incomplete observations. *J Am Statist Assoc* **53**: 457–481

Longo DL, Duffey PL, DeVita VJ, Wesley MN, Hubbard SM and Young RC (1991) The calculation of actual or received dose intensity: a comparison of published methods. *J Clin Oncol* **9**: 2042–2051

Mouridsen HT, Rose C, Brincker H, Thorpe SM, Rank F, Fischerman K and Andersen KW (1984) Adjuvant systemic therapy in high-risk breast cancer: the Danish Breast Cancer Cooperative Group's trials of cyclophosphamide or CMF in premenopausal and tamoxifen in postmenopausal patients. *Recent Results Cancer Res* **96**: 117–128

Peto R, Pike M and Armitage P (1977) Dosing and analysis of randomized clinical trials requiring prolonged observation of each patient. II. Analysis and examples. *Br J Cancer* **35**: 1–39

Pronzato P, Campora E, Amoroso D, Bertelli G, Botto F, Conte PF, Sertoli MR and Rosso R (1989) Impact of administration-related factors on outcome of adjuvant chemotherapy for primary breast cancer. *Am J Clin Oncol* **12**: 481–485

Redmond C, Fisher B and Wieand HS (1983) The methodologic dilemma in retrospectively correlating the amount of chemotherapy received in adjuvant therapy protocols with disease-free survival. *Cancer Treatment Rep* **67**: 519–526

Rodriguez KR, Hortobagyi GN, Buzdar AU and Blumenschein GR (1981) Combination chemotherapy for breast cancer metastatic to bone marrow. *Cancer* **48**: 227–232

Saarto T, Blomqvist C, Rissanen P, Auvinen A and Elomaa I (1997) Haematological toxicity: a marker of adjuvant chemotherapy efficacy in stage II and III breast cancer. *Br J Cancer* **75**: 301–305

Senn H, Jungi W and Amgewerd R (1984) Adjuvant chemoimmunotherapy with LMF + BCG in node-negative and node positive breast cancer. 8 year results. *Recent Results Cancer Res* **96**: 90–101

Tormey D, Gelman R and Falkson G (1983) Prospective evaluation of rotating chemotherapy in advanced breast cancer. An Eastern Cooperative Oncology Group Trial. *Am J Clin Oncol* **6**: 1–18

Velez-Garcia E, Carpenter J and Moore M (1987) Postsurgical adjuvant chemotherapy with or without radiotherapy in women with breast cancer and positive axillary nodes: Progress report of Southeastern Cancer Study Group (SEG) trial. *Adjuvant Ther Cancer* **V**: 347–355

Wood WC, Budman DR, Korzun AH, Cooper MR, Younger J, Hart RD, Moore A, Ellerton JA, Norton L, Ferree CR, Colangelo Ballow A, Frei III E and Henderson C (1994) Dose and dose intensity of adjuvant chemotherapy for stage II, node-positive breast carcinoma *N Engl J Med* **330**: 1253–1259

 *© 1999 Cancer Research Campaign*
